# Automated psychological therapy using virtual reality (VR) for patients with persecutory delusions: study protocol for a single-blind parallel-group randomised controlled trial (THRIVE)

**DOI:** 10.1186/s13063-019-3198-6

**Published:** 2019-01-29

**Authors:** Daniel Freeman, Rachel Lister, Felicity Waite, Ly-Mee Yu, Mel Slater, Graham Dunn, David Clark

**Affiliations:** 1 Department of Psychiatry, University of Oxford, Warneford Hospital, Oxford, OX3 7JX UK; 2 Oxford Health NHS Foundation Trust, Oxford, UK; 3 NIHR Oxford Health Biomedical Research Centre, Oxford, UK; 4 Primary Care Clinical Trials Unit, Nuffield Department of Primary care Health Sciences, University of Oxford, Oxford, UK; 5 Department of Clinical Psychology and Psychobiology, Faculty of Psychology, University of Barcelona, Barcelona, Spain; 6 Division of Population Health, Health Services Research & Primary Care, University of Manchester, Manchester, UK; 7 Department of Experimental Psychology, University of Oxford, Oxford, UK

**Keywords:** Psychosis, Schizophrenia, Virtual reality (VR), Cognitive therapy, Automated delivery

## Abstract

**Background:**

Persecutory delusions are a major psychiatric problem and are associated with a wide range of adverse outcomes. Our theoretical model views these delusions as unfounded threat beliefs which persist due to defence behaviours (e.g. avoidance) that prevent disconfirmatory evidence being processed. The treatment implications are that patients need to (1) go into feared situations and (2) not use defence behaviours. This enables relearning of safety and hence paranoia diminution. However, this is very difficult for patients due to their severe anxiety. A solution is to use virtual reality (VR) social situations, which are graded in difficulty and which patients find much easier to enter. We have now automated the provision of cognitive therapy within VR using an avatar coach, so that a therapist is not required and the treatment is scalable. In the THRIVE trial, the automated VR cognitive treatment will be tested against a VR control condition. It will contribute to our wider programme of work developing VR for patients with psychosis.

**Methods:**

Patients with persistent persecutory delusions in the context of non-affective psychosis will be randomised (1:1) to the automated VR cognitive treatment or VR mental relaxation (control condition). Each VR treatment will comprise approximately four sessions of 30 min. Standard care will remain as usual in both groups. Assessments will be carried out at 0, 2, 4 (post treatment), 8, 16, and 24 weeks by a researcher blind to treatment allocation. The primary outcome is degree of conviction in the persecutory delusion (primary endpoint 4 weeks). Effect sizes will be re-established by an interim analysis of 30 patients. If the interim effect size suggests that the treatment is worth pursuing (*d* > 0.1), then the trial will go on to test 90 patients in total. Secondary outcomes include real world distress, activity levels, suicidal ideation, and quality of life. Mediation will also be tested. All main analyses will follow the intention-to-treat principle. The trial is funded by the Medical Research Council Developmental Pathway Funding Scheme.

**Discussion:**

The trial will provide the first test of automated cognitive therapy within VR for patients with psychosis. The treatment is potentially highly scalable for treatment services.

**Trial registration:**

ISRCTN, ISRCTN12497310. Registered on 14 August 2018.

**Electronic supplementary material:**

The online version of this article (10.1186/s13063-019-3198-6) contains supplementary material, which is available to authorized users.

## Background

Persecutory delusions are a person’s unfounded beliefs that other people are intending to harm that person (e.g. ‘People know what I’m thinking and are trying to kill me’). Approximately 70% of patients with schizophrenia have this type of psychotic experience [1], which typically leads to social withdrawal, suicidal ideation, and hospital admission [2–5]. Almost half of individuals with persecutory delusions have levels of psychological well-being in the lowest 2% of the general population [6]. However, more than half of patients with psychotic conditions such as schizophrenia do not respond adequately to current treatments [7, 8]. The median treatment effect size for antipsychotic medications is 0.44 [9], while standard first generation psychological therapy has an effect size of approximately 0.36 [10]. These are small to moderate effects for a patient group that has severe problems. Treatment needs to be significantly improved. We take a translational approach, allied to technological advances, for new treatment development for persecutory delusions.

Our cognitive model conceptualises persecutory delusions as unfounded threat beliefs maintained by defence (‘safety-seeking’) behaviours [11]. Patients, for example, often avoid other people, avert their gaze, and remain vigilant, attributing the absence of harm to the use of such actions instead of the inaccuracy of the threat beliefs (e.g. ‘The reason I wasn’t attacked was because I kept away from people’). The target mechanism for successful treatment is for patients to relearn that they are safe (in order to counteract the threat belief). This involves re-evaluating the threat beliefs. However, many patients with persecutory delusions find it too difficult to enter their feared situations and drop their defences because of the intolerable anxiety generated. When they are admitted to psychiatric hospital, opportunities for learning of safety in everyday situations are even more highly restricted.

We have argued that virtual reality ([VR] interactive computer-generated environments) may provide a powerful treatment approach for patients to enable the learning of safety that can reduce delusions [12, 13]. In a controlled environment with a graded approach, VR allows individuals to repeatedly experience the situations they find difficult and potentially enable new learning. Patients are much more likely to test out their fear expectations in VR because they know it is a simulation, but the learning that they achieve then transfers to the real world. VR treatments have the potential to be faster, more efficacious, and appealing to patients than traditional face-to-face approaches. Initially we showed that VR can be used to assess paranoia [14] and understand the causes [15], and that it is safe to use with patients with persecutory delusions [16]. We then conducted a first test of VR to treat persecutory delusions [17]. In this pilot study, 30 patients with persecutory delusions (despite taking antipsychotic medication) in the context of schizophrenia spectrum disorders were randomised to VR cognitive treatment (VRCT) (testing threat beliefs while dropping defence behaviours) or VR exposure therapy (VRET). We used 30 min in graded VR environments (a train and lift scenario) with psychological advice provided by a therapist. Before and after VR, the patients completed a real world situation task that they found difficult (e.g. going into a shop), and the delusion was assessed. In comparison to VRET, VRCT led to large reductions in delusions (*d* = 1.3) and in distress in the real world (*d* = 0.8). In sum, there were large improvements in delusions and a transfer of benefits to the real world, even compared to another effective treatment approach [17]. Recently, a randomised controlled trial of more than 100 patients with psychosis showed that 16 sessions with a therapist using VR also produced large reductions in paranoia compared to usual care (*d* = 1.5) [18].

Previous uses of VR for mental health problems have all used VR to elicit symptoms; a therapist then provides the psychological therapy [13]. Hence, VR has been used as an aid for therapists. However, we have recently shown that it is possible to automate the provision of psychological therapy with VR. We tested the efficacy of an automated cognitive intervention for fear of heights guided by an avatar virtual coach (animated using the motion and voice capture of an actor) in VR and delivered with the latest consumer equipment [19]. The effect sizes of the VR treatment were very large (Cohen’s *d* = 2.0; the number needed to treat [NNT] to reduce fear by at least 50% was 1.3). In our new trial (THRIVE) we will test a four-session automated cognitive treatment in VR (VRCT) for patients with persecutory delusions. This is part of a larger programme of work by our team — called ‘gameChange’ (www.gameChangeVR.com) — developing the use of VR for patients with psychosis.

VRCT comprises two key elements: (1) entering the feared situations and (2) not using defence (‘safety-seeking’) behaviours. By testing against exposure, our pilot actually controlled for the first treatment element of entering the feared situations. This means that the ‘in toto’ effect of the cognitive treatment will have been underestimated. We will now estimate treatment effects by comparing against a highly credible VR control treatment (mental relaxation training) that does not use either of the key cognitive elements. The primary hypothesis is that:

1. Compared to the control condition, VRCT will lead to a reduction in delusional conviction (post treatment, 4 weeks).

The secondary hypotheses are that:

2. Compared to the control condition, VRCT will lead to reductions in distress in real world situations, overall paranoia, delusion severity, and suicidal ideation, and increases in activity, well-being, and quality of life (post treatment, 4 weeks).
3. The benefits of VRCT will be maintained over time.
4. Change in delusion conviction will be mediated by changes in beliefs about safety and use of defence behaviours.

## Methods

**Fig. 1:**
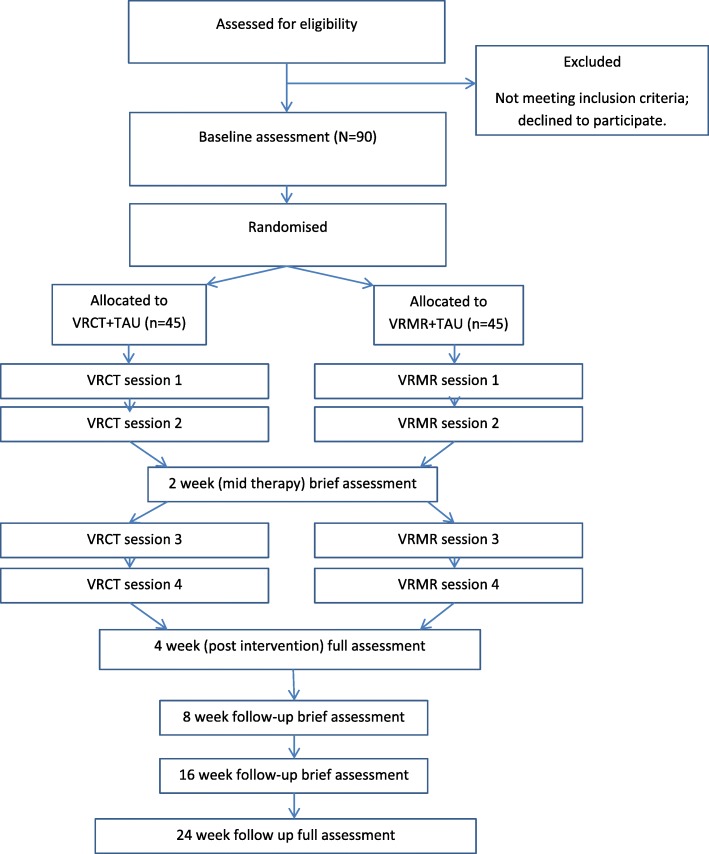
Trial flow diagram

**Fig. 2:**
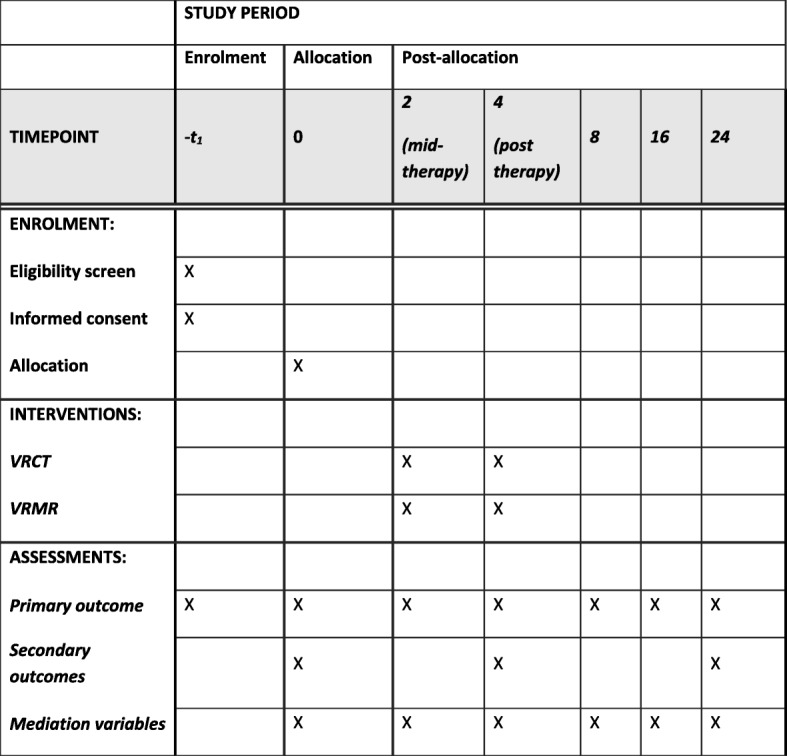
The schedule of enrolment, interventions, and assessments (SPIRIT 2013 guidelines)

The trial is called THRIVE (THerapeutic Realistic Immersive Virtual Environments). The design is a parallel-group randomised controlled trial with single-blind assessment to test whether the automated VRCT will reduce persecutory delusions more effectively than VR mental relaxation (VRMR). Standard care will be measured but remain as usual in both groups. Assessments will be carried out at 0, 2, 4 (post treatment), 8, 16, and 24 weeks by a researcher blind to treatment allocation (see Fig. 1 for the trial flow diagram). The repeated follow-up points are used to enable the observation of the time course of any treatment effects. The trial has received approvals from the National Health Service (NHS) Health Research Authority and a notice of no objection for a trial of a medical device from the UK’s Medicines and Healthcare products Regulatory Agency (MHRA), and it has been prospectively registered (ISRCTN12497310; http://www.isrctn.com/ISRCTN12497310). Written informed consent will be obtained from all trial participants. There is a trial Data Monitoring and Ethics Committee (DMEC). The trial is also supported by a Patient Advisory Group (PAG) run for our research group by the McPin Foundation. The basic trial methods of enrolment, interventions, and assessments are summarised in Fig. 2. The Standard Protocol Items: Recommendations for Interventional Trials (SPIRIT) checklist is provided as Additional file 1.

### Participants

Referrals will be sought from Oxford Health NHS Foundation Trust and neighbouring NHS Trusts (e.g. Northamptonshire Healthcare NHS Foundation Trust, Berkshire Healthcare NHS Foundation Trust, the Milton Keynes locality of Central North West London NHS Foundation Trust). The inclusion criteria are as follows: aged 16 years or older; persistent (at least 3 months) persecutory delusion (as defined by Freeman and Garety [20]) held with at least 50% conviction; reporting feeling threatened when with other people; diagnosis of schizophrenia spectrum psychosis (non-affective psychosis). The exclusion criteria are the following: primary diagnosis of alcohol or substance disorder; photosensitive epilepsy; significant visual, auditory, or balance impairment; current receipt of another psychological therapy; insufficient comprehension of English; being treated in forensic settings; a diagnosis of organic syndrome; significant learning disability; current active suicidal plans. (These exclusion criteria are checked with the clinical team and with the patient.) A participant may also not enter the trial if there is another factor that in the judgement of the investigator would preclude the participant from providing informed consent or from safely engaging in the trial procedures. Reasons for exclusion will be recorded in line with Consolidated Standards of Reporting Trials (CONSORT) guidelines.

### Randomisation and blinding

Randomisation will occur after completion of the baseline assessment. Allocation to the two conditions will be in a 1:1 ratio. Randomisation will be carried out by an online system designed by the University of Oxford Primary Care Clinical Trials Unit. Randomisation using a permuted blocks algorithm, with randomly varying block size, will be stratified by severity of delusion: moderate (50–75% conviction)/high (76% + conviction).

The trial assessors will be blind to group allocation, but the patients and trial therapists will know the allocations. As all participants receive a four-session VR treatment (i.e. delivery of treatment is the same), it will be easier to conceal from a research assistant which treatment type a patient is receiving. The trial therapists will inform patients of the randomisation outcome, so that the research assessors remain blind to group allocation. Precautionary strategies to prevent breaking of the blind include patients being reminded by the assessor not to talk about treatment allocation and, after the initial assessment, the assessor not looking at a patient’s clinical notes. If an allocation is revealed between assessment sessions, this will be recorded by the trial co-ordinator, and re-blinding will occur using another assessor.

### Baseline assessments

Basic demographic and clinical data will be collected (e.g. age, gender, ethnicity, clinical diagnosis).

The primary outcome measure will be degree of conviction in the persecutory delusion (using a 0–100% scale) assessed within the Psychotic Symptom Rating Scale-Delusions (PSYRATS) [21]. A dimensional score (0–100%) will be used, as in the Feeling Safe Study [22].

There are a number of secondary outcome measures. As in our pilot, a real world behavioural test will assess distress in real situations [17]. Overall paranoia will be assessed with the Green et al. Paranoid Thoughts Scale (GPTS) [23] and delusion severity with the PSYRATS [21]. Suicidal ideation will be assessed with the Columbia-Suicide Severity Rating Scale [24]. Activity levels (step count) will be assessed using actigraphy, complemented with a time budget assessing meaningful activity [25]. The EuroQol Five Dimensions Five Levels (EQ-5D-5 L) (http://www.euroqol.org/) will assess quality of life. Well-being will be assessed with the Warwick-Edinburgh Mental Wellbeing Scale (WEMWBS) [26], the Questionnaire about the Process of Recovery (QPR) [27], and an adapted version of the CHOICE [28]. We will record service use, and other relevant health economic data, using an adapted version of the Economic Patient Questionnaire (EPQ) [29] that includes questions from the Client Service Receipt Inventory [30].

For tests of treatment mediation, we will assess use of defence behaviours with the Safety Behaviours Questionnaire (SBQ) [31] and strength of beliefs about safety (using a visual analogue scale as in Freeman and colleagues [22]).

All the preceding measures are completed at the main assessment time points: 0, 4, and 24 weeks. The exceptions are the behavioural test, assessing distress in real situations, which will only be repeated at the 4 weeks assessment, and the health economic data questionnaire, which is re-assessed at 24 weeks. The primary outcome (delusional conviction), delusion measures (PSYRATS, GPTS), and mediation measures (SBQ and safety belief) will be tested at all assessment points (0, 2, 4, 8, 16, 24 weeks).

The credibility of the treatments will be assessed with the Credibility/Expectancy Questionnaire [32] at the first treatment session.

### Treatments

A mental health professional will be in the room when the two treatments are given. This person will help the patient put on the VR headset and start the appropriate programme. The applications will run through the Steam software application on a laptop computer connected to an HTC Vive headset and accessories (two handheld controllers and two ‘lighthouse’ sensors, set up in corners of the room). All technical requirements will be as per HTC Vive requirements. The accessory hardware and software are already commercially available and have not been modified for the trial. The number of sessions and treatment time will be recorded. Participants are free to withdraw from treatment at any point.

#### VR cognitive treatment (VRCT)

This treatment aims for patients to test their fear expectations around other people in order to relearn safety and hence diminish the delusion. The treatment is not designed as exposure therapy (participants are not asked to remain in situations until anxiety reduces) but as repeated behavioural experiment tests (enabling patients to learn that they are safer than they had thought). The treatment is designed to be delivered in approximately four sessions of 30 min. However, participants can proceed at their own pace, meaning that a smaller or greater number of sessions is allowed. The VR treatment is set in a virtual shopping centre. A virtual coach guides the person through the treatment, including encouraging the dropping of defence behaviours, and elicits feedback to tailor the progression of the treatment. At the beginning of the first session, the virtual coach, in a virtual office in the shopping centre, explains the rationale behind the treatment, and the participant selects which one of four VR situations they would like to begin in. The four VR scenarios set in the shopping centre are a café, a lift, a central area, and a clothes shop. Each scenario has five degrees of difficulty (e.g. the number and proximity of people in the social situation increases), and participants work their way through each level of difficulty. The participant typically stands and is able to walk a few paces in the scenarios. The participant can choose a different scenario in each session or repeat a previous situation. Throughout the sessions, participants’ responses to questions from the virtual coach are given by means of a virtual watch. Belief ratings are repeated within VR at the end of each treatment session. In order for the purpose of the treatment to be clearer for patients in the trial, it will be described as ‘VR Confidence Building’, since the goal is to increase confidence in everyday situations around other people.

#### VR mental relaxation (VRMR)

This treatment aims for patients to feel calmer using relaxation techniques in order to be less anxious about other people. It is explained to participants that a helpful way to counteract a fear is to have a calm mind. In each session, participants will choose from a selection of calm VR environments (e.g. a beach, the countryside, a forest, and a lake) taken from a commercially available VR relaxation programme in which they will practice a number of relaxation techniques. These are non-social environments. Within a session a participant can choose to change the VR environment. Typically the person will sit during the relaxation exercises. The number of sessions and time in VR will be equivalent to those for VRCT.

### Adverse events

A trial standard operational procedure has been written for adverse events. We will record the occurrence of any serious adverse events (SAEs) reported to us and also check each patient’s medical notes at the end of their participation in the trial. An adverse event is defined by the ISO14155:2011 guidelines for medical device trials as serious if it (1) results in death, (2) is a life-threatening illness or injury, (3) requires (voluntary or involuntary) hospitalisation or prolongation of existing hospitalisation, (4) results in persistent or significant disability or incapacity or (5) medical or surgical intervention required to prevent any of the above, (6) leads to foetal distress, foetal death or consists of a congenital anomaly or birth defect, or (7) is otherwise considered medically significant by the investigator.

Life threatening in the definition of an SAE refers to an event in which the subject was at risk of death at the time of the event; it does not refer to an event that hypothetically might have caused death if it were more severe. A planned hospitalization for a pre-existing condition, without a serious deterioration in health, is not considered to be an SAE. The sorts of SAEs that can happen to this participant group include deaths, suicide attempts, serious violent incidents, and admissions to hospital.

We will also record any adverse device effects from the VR treatment, which includes adverse events resulting from insufficient or inadequate instructions for use, deployment, installation, or operation, or any malfunction of the software. It also includes any event resulting from user error or intentional misuse.

### Analysis

A full statistical analysis plan will be written prior to any analysis being undertaken. We will report data in line with the CONSORT 2010 Statement showing attrition rates and loss to follow-up. All analyses will be carried out using the intention-to-treat principle with data from all participants included in the analysis including those who do not complete therapy. Every effort will be made to follow up all participants in both arms for research assessments.

Descriptive statistics within each randomised group will be presented for baseline values. These will include counts and percentages for binary and categorical variables and means and standard deviations, or medians with lower and upper quartiles, for continuous variables, along with minimum and maximum values and counts of missing values. There will be no tests of statistical significance or confidence intervals for differences between randomised groups on any baseline variable.

Based on the pilot test against exposure [17], we expect at least a 20% reduction in delusional conviction (effect size = 1.0) with VRCT compared to VRMR. This is a conservatively lower effect size expectation than the first pilot. Nonetheless, we recognise that an even lower effect size (*d* = 0.75, reflecting a 15% reduction in conviction) for delusions would still be of interest to pursue, and thus we power the full trial (*n* = 90) on this basis. A trial with 45 participants in each arm (allowing up to 15% loss to follow-up) will have approximately 90% power to detect a statistically significant treatment effect at 4 weeks, using an independent groups *t* test and a significance level of 0.05, if the true standardised effect size is 0.75. Following guidelines for good practice, interim analysis will provide simple descriptive statistics and an initial estimate of the 95% confidence interval for the treatment effect. This interim analysis of the week 4 data after 30 participants will provide an estimate of conditional power (i.e. power given the data obtained so far) [33, 34]. We will stop the trial if the interim estimate of effect size, *d*, is 0.1 or lower, implying that the conditional power of the full trial, based on the interim results and the hypothesised effect size of 0.75, would be 60% or lower. If it were assumed that the treatment effect seen in the pilot would continue throughout the rest of the trial, then the conditional power would be as low as 3%.

The primary hypothesis is for change in conviction in the persecutory delusions (using a 0–100% scale) at 4 weeks. Additionally, repeated measures are also assessed at the 4 weeks point and again at 8, 16, and 24 weeks. Random or mixed effects models will be fitted to the repeated measures to estimate treatment effects. The mixed effect models will include the outcome as the response variable, time point, randomised group, and baseline score as fixed effects, and a patient-specific random intercept. An interaction between time and randomised group will be fitted as a fixed effect to allow estimation of treatment effect at all time points. *P* < 0.05 will be used as the level of statistical significance.

The mediation analysis will investigate putative mediational factors (safety behaviours and safety beliefs) using modern causal inference methods [35]. This involves using parametric regression models to test for mediation of VRCT on outcome through the putative mediators. Analyses will adjust for baseline measures of the mediator, outcomes, and possible measured confounders. We will include repeated measurement of mediators and outcomes to account for classical measurement error and baseline confounding.

## Discussion

Over the past 25 years VR has been applied with promising results to the understanding and treatment of mental health disorders. Its use in treatment has been as an aid for therapists, typically delivering exposure treatments; therefore, the potential clinical benefits of VR have still relied on the abilities of the clinician in the room. The THRIVE trial will provide a first test of an automated delivery of psychological therapy using VR for patients with psychosis. Therefore, patients can all experience the same standard of psychological therapy. The therapy delivered in VR is built upon the principle that paranoia diminishes when expectations of harm from other people are put to the test. The comparison in the trial is against similar time in VR but encouraging the reduction of fear via relaxation techniques (i.e. using a different psychological intervention and mechanism). There are repeated follow-up assessments of the main outcome to enable the persistence of any clinical gains to be tracked over time and in order to support mediation tests. The THRIVE trial also provides a first opportunity to learn lessons about the implementation of automated psychological therapy using consumer equipment within UK NHS mental health trusts. It will inform our new National Institute for Health Research (NIHR) i4i mental health challenge award programme of work, gameChange (www.gameChangeVR.com). In gameChange, the VR treatment has been completely redesigned, reprogrammed, lengthened, and made suitable for any patient with psychosis who is fearful of social situations. It will be tested in a large multi-centre trial starting later in 2019. The gameChange project also systematically examines implementation issues for automated VR treatment in psychosis services [36].

### Trial status

The trial started patient recruitment in October 2018. Outcome results are expected at the end of 2020. A trial paper with the results should be submitted for publication around the beginning of 2021.

## Additional file

Additional file 1: SPIRIT 2013 checklist: recommended items to address in a clinical trial protocol and related documents. (DOC 118 kb)

## Abbreviations

CHOICE: CHoice of Outcome In Cbt for psychosEs
CONSORT: Consolidated Standards of Reporting Trials
CSRI: Client Service Receipt Inventory
DMEC: Data Monitoring and Ethics Committee
EPQ: Economic Patient Questionnaire
EQ-5D-5 L: EuroQol Five Dimensions Five Levels
GPTS: Green et al. Paranoid Thoughts Scale
MHRA: Medicines and Healthcare products Regulatory Agency
MRC: Medical Research Council
NHS: National Health Service
NNT: Number needed to treat
PAG: Patient Advisory Group
PSYRATS: Psychotic Symptom Rating Scale
SAE: Serious adverse event
SBQ: Safety Behaviours Questionnaire
SPIRIT: Standard Protocol Items: Recommendations for Interventional Trials
TAU: Treatment as usual
THRIVE: THerapeutic Realistic Immersive Virtual Environments
VR: Virtual reality
VRCT: Virtual reality cognitive treatment
VRMR: Virtual reality mental relaxation
WEMWBS: Warwick-Edinburgh Mental Wellbeing Scale
